# Crosstalk between mitotic reassembly and repair of the nuclear envelope

**DOI:** 10.1080/19491034.2024.2352203

**Published:** 2024-05-23

**Authors:** Yohei Kono, Takeshi Shimi

**Affiliations:** a Nano Life Science Institute (WPI-NanoLSI), Kanazawa University, Kanazawa, Japan; b Cell Biology Center, Institute of Innovative Research, Tokyo Institute of Technology, Yokohama, Japan

**Keywords:** BAF, ESCRT-III, lamin, lamina, NPC, nuclear envelope, nuclear envelope reassembly, nuclear envelope rupture

## Abstract

In eukaryotic cells, the nuclear envelope (NE) is a membrane partition between the nucleus and the cytoplasm to compartmentalize nuclear contents. It plays an important role in facilitating nuclear functions including transcription, DNA replication and repair. In mammalian cells, the NE breaks down and then reforms during cell division, and in interphase it is restored shortly after the NE rupture induced by mechanical force. In this way, the partitioning effect is regulated through dynamic processes throughout the cell cycle. A failure in rebuilding the NE structure triggers the mixing of nuclear and cytoplasmic contents, leading to catastrophic consequences for the nuclear functions. Whereas the precise details of molecular mechanisms for NE reformation during cell division and NE restoration in interphase are still being investigated, here, we mostly focus on mammalian cells to describe key aspects that have been identified and to discuss the crosstalk between them.

## Structure and organization of the NE

**Figure 1.**
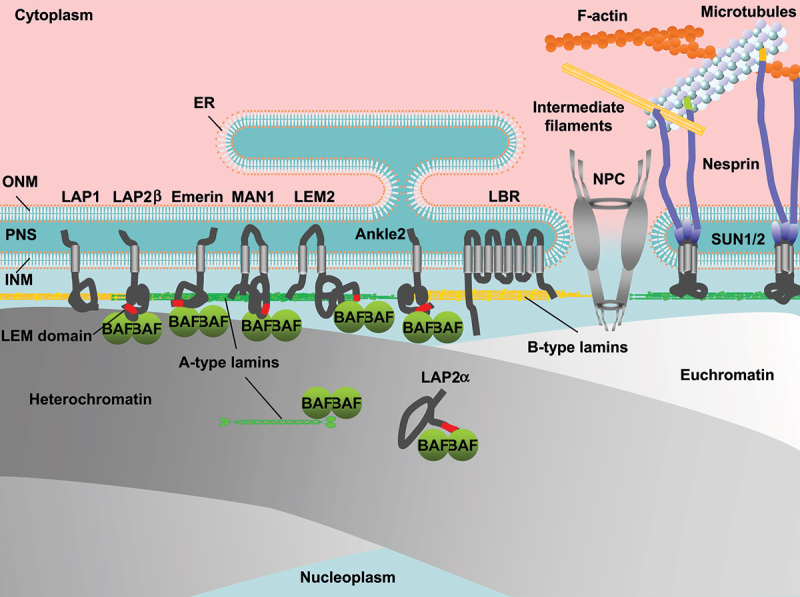
Schematic depiction of the nuclear periphery including the nuclear envelope (NE), chromatin and the cytoskeleton. Nuclear membranes, separating between the nucleoplasm and the cytoplasm consist of the inner and outer nuclear membranes (INM, ONM) and are gapped with the perinuclear space (PNS). The ONM continues with the endoplasmic reticulum (ER) on the cytoplasmic side whereas the nuclear side of the INM is underlined with the nuclear lamina (NL), the major structural components of which are A- and B-type lamins. The INM is inserted with INM proteins including LAP1, LAP2β, emerin, MAN1, LEM2, Ankle2, LBR and SUN1/2. LAP2α interacts with A-type lamins in the nucleoplasm. BAF dimers bind to A-type lamins and LEM-domains. Lamins and INM proteins associate with heterochromatin at the nuclear periphery. SUN1/2 dimers interact with nesprins in the PNS to form the LINC complex, which mediate nuclear membranes and cytoskeletal systems. The nuclear pore complex (NPC) penetrates INM and the ONM, and the structural components nucleoporins (nups) interact with euchromatin.

In eukaryotic cell nuclei, the genome is surrounded with the nuclear envelope (NE) that consists of the double phospholipid bilayers of the inner and outer nuclear membranes (INM, ONM) and NE-associated proteins (Figure 1). In mammalian cells, the nuclear lamina (NL) underlines the nuclear side of the INM to maintain the nuclear shape, size and stiffness [1–4]. The major structural determinants of the NL are type-V intermediate filaments proteins, the nuclear lamins. They are subdivided into A-types (lamins A (LA) and C (LC)) and B-types (lamins B1 (LB1) and B2 (LB2)). Lamins assembles into a filament with a diameter of ~3.5 nm, and these lamin filaments are nonrandomly distributed to form a fiber of meshworks in the NL with a thickness of ~14 nm [5,6]. The INM is inserted with a group of proteins with a transmembrane domain (TM) that associate with the NL such as lamina-associated polypeptide (LAP) 1, LAP2/Emerin/MAN1 (LEM)-domain proteins including LAP2β, emerin, MAN1, LEM2 and Ankle2, lamin B receptor (LBR) and Sad1p/UNC-84 (SUN) domain proteins SUN1/2. Another LEM-domain protein LAP2α does not have the TM but interacts with A-type lamins in the nucleoplasm [7]. Barrier-to-autointegration factor (BAF) forms dimers and interact with chromatin [8,9]. BAF dimers also bind to A-type lamins and LEM-domain proteins through the immunoglobulin-like fold (Ig-fold) domain and the LEM domain, respectively [9–11]. Lamins and INM proteins associate with heterochromatin at the nuclear periphery [12]. The ONM structurally continues with the endoplasmic reticulum (ER) [13] on the cytoplasmic side and is inserted with another group of proteins with the TM including nesprins [14,15]. Nesprins interacts with SUN1/2 dimers through the klarsicht/ANC-1/SYNE homology (KASH) domain inside the lumen between the INM and the ONM called the perinuclear space (PNS), forming the linker of nucleoskeleton and cytoskeleton (LINC) complex to connect the INM and the ONM with cytoskeletal systems including F-actin, microtubules and intermediate filaments [16]. Nuclear pore complexes (NPCs) are embedded in annular junctions between the INM and the ONM to facilitate nucleo-cytoplasmic transport [17] whereas bound to lamin filaments in the NL [18,19]. The overall structure is a cylindrical shape with a diameter of ~120 nm and contains more than 30 proteins known as nucleoporins (Nups) [20–25]. Nups interact with euchromatin on the nuclear side of NPCs for gene activation [26,27].

## Mitotic NE disassembly

In mitosis, a mother cell divides into two daughter cells that usually have the same number of chromosomes as the original one. There are ‘closed’ and ‘open’ mitosis among different organisms [28]. Closed mitosis occurs without NE breakdown (NEBD), and chromosomes remain inside the nucleus. In contrast, chromosomes become exposed to the cytoplasm in open mitosis (hereinafter called mitosis) as nuclear compartments including the NE disintegrates during NEBD. Then, these compartments are reformed on daughter chromosomes by the exit. In mammalian cells, as the cell enters prophase, the chromatin in the nucleus begins to condense into chromosomes. The NE starts disassembling at the onset of NEBD, subsequentially followed by the retraction of nuclear membranes into the ER [29–31]. In prometaphase, NEBD is complete as the NL and NPCs disassemble, followed by the fragmentation of nuclear membranes. Then, microtubules attach to kinetochores and chromosomes congress to the metaphase plate. The initiation of NE disassembly is coupled to the phosphorylation of NE components including lamins [32–35], BAF [9,36], INM and ONM proteins [7,37–40] and Nups [41–44] by cyclin-dependent kinase 1 (CDK1), protein kinase C (PKC), vaccinia-related kinase 1 (VRK1), aurora kinases, polo-like kinase 1 (PLK1) and never in mitosis gene A (NIMA)-related kinases (NEK) [45]. Before NEBD starts in a large scale, a CDK1-cyclin B1 complex is actively transported to the nucleus in a cyclin A2-dependent manner so that CDK1 can access to the phosphorylation sites of NE components inside the nucleus [46–49]. Supporting this idea, the NE is found permeable to cytoplasmic macromolecules before NEBD as some of phosphorylated Nups are disassembled from NPCs to form holes in the NE [50–52]. The NE might be also torn by the mechanical tension with the spindle microtubules and dynein motors as dynein/dynactin component p62 complex is translocated to the ONM just before NEBD [53]. Elongating microtubules from spindle asters apparently push on the nuclear surface and eventually penetrate the NE [54] and the holes of the NE expand until the NE becomes fragmented [53,55]. The disassociation of NE components from the NE differs in timing. For example, the hyperphosphorylation of Nup98 by CDK1 and NEK is the initial step of the NPC disassembly [43]. Simultaneously or subsequently, CDK1 might also phosphorylate the Nup107–160 subcomplex, an important building block of the NPC, and embryonic large molecule derived from yolk sac (ELYS) for their disassociations from the NPC [56,57]. CDK1 and PKC concertedly phosphorylate the serines of lamins which are located just outside the N-terminal and C-terminal ends of the rod domain, resulting in the depolymerization of the head-tail polymers in the NL [32,33,58–60]. Then, the depolymerized LA/C appears to start dissociating from the NL and diffuse into the nucleoplasm, followed by the fragmentation of the NL with the depolymerized LB1 and LB2 at the prophase/prometaphase transition [54].

## Mitotic NE reassembly

Whereas kinetochore microtubules get shortened to pull the sister chromatids of each mitotic chromosome apart during the metaphase-anaphase transition and telophase until the chromosomes reach the poles of the cell, the NE reforms around daughter chromosomes. For mitotic NE reassembly, phosphorylated forms of NE components are directed to the surface of the chromosomes through various pathways [61,62]. Processes of the NE reassembly are linked to the dephosphorylation of these NE components [63]. At the entry of mitosis, protein phosphatase (PP) 1 and PP2A are inactivated through CDK1-mediated phosphorylation [64–66]. As the CDK1 expression level declines in the late stages, reactivating PP1 and PP2A [67,68], PP1 could remove phosphates from phosphorylated NE components at sites of NE reformation [69]. PP1 directly binds to an ER/NE membrane integrated protein A-kinase anchoring protein 149 (AKAP149) to remain anchored at the NE during G1 phase until it is released from AKAP149 upon S phase entry through the phosphorylation [69–71]. PP1 is also recruited to the chromosome surface in anaphase by its binding to Repo-man [72]. Repo-man also binds to Importin β to promote the recruitment, and the phosphorylation of Repo-man by the CDK1-cyclin B1 complex inhibit the binding [73]. In this way, PP1 localization at the NE allows the temporal and spatial regulation of mitotic NE reassembly.

**Figure 2.**
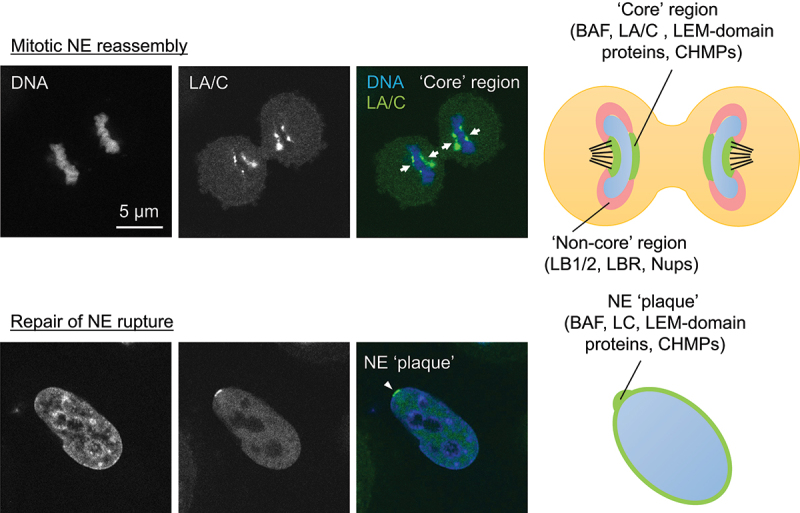
Representative single confocal section images and schematic depictions of the core region and the NE plaque. HeLa cells expressing sfGFP-DARPin-LA6 [82] were fixed with 4% PFA, followed by permeabilization with 0.1% Triton X-100 and DNA staining with Hoechst 33342 at telophase (top) and after the induction of NE rupture by 405-nm laser-microirradiation (bottom). Arrow: core region. Arrowhead: NE plaque. Bar: 5 μm.

**Figure 3.**
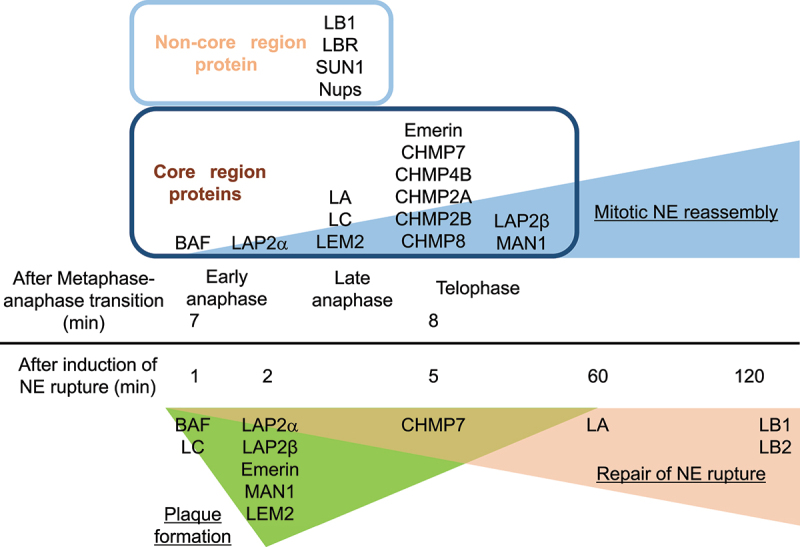
Sequential order of NE components in accumulation at or localization to the core region and the non-core region, and the NE plaque. The order and time (min) of the indicated proteins in mitotic NE reassembly (top) and repair of NE rupture (bottom).

**Figure 4.**
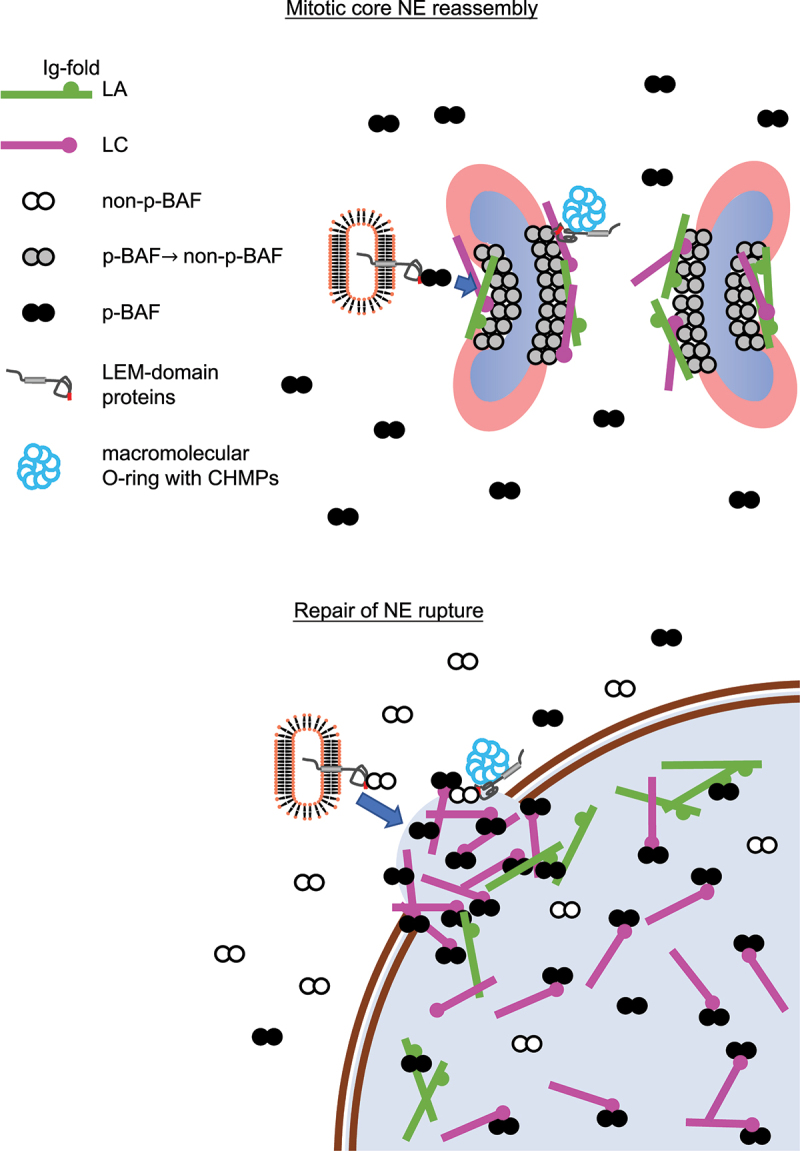
Schematic diagram of NE-component accumulation at or localization to the core region and the NE plaque. (Top) during mitosis, LA/C and LEM-domain proteins are recruited to the core region with BAF, followed by the accumulation of CHMPs to form a macromolecular O-ring. (Bottom) Phosphorylated BAF (p-BAF) recruits LC from the nucleoplasm to form the NE plaque on the surface of chromatin exposed to the cytoplasm at the opening area of a rupture site.

BAF accumulates at the central region of chromosomes close to kinetochores and telomeres during late anaphase and telophase, designated as the ‘core’ region (~5 min) immediately followed by LAP2α accumulation [62,74] (Figure 3). Then, it sequentially recruits LA/C and LEM-domain containing INM proteins including LAP2β, Emerin, MAN1, LEM2 and Ankle2 to the core region [62,74,75]. The BAF accumulation requires the DNA-binding ability [8,10] as BAF can bind to double-stranded (ds) DNA in a sequence-independent manner [8,76–78] and form a dimer or even an oligomer with dsDNA using two pairs of helix-hairpin-helix motifs located on opposite surfaces of the dimer without changing its conformation [79]. The dephosphorylation of BAF might be also required for its accumulation (Figure 4). To support this idea, VRK1 efficiently phosphorylates the N-terminal residues Ser-4 and/or Thr-2/Thr-3 of BAF inside the nucleus [9,36,80] (Figure 4). After the phosphorylated form is released from chromatin during NEBD [81], it could bind to A-type lamins and LEM-domain proteins in the cytoplasm for the recruitment to the core region through the Ig-fold domain and the LEM domain, respectively (Figure 2) [8,10,62,74,78,83–89], and later it becomes non-phosphorylated at the NE [75,80,90] (Figure 4). In addition, Ankle2 targets PP2A to BAF and associates with VRK1 to inhibit its kinase activity, which enhances BAF dephosphorylation [75]. Therefore, the kinase and phosphatase could be involved in regulating BAF accumulation at the core region. BAF and LA/C are mutually dependent on each other for the accumulation to the core region [74,91,92], which is likely to be mediated by BAF binding to the Ig-fold domain and facilitated by the regulation of BAF phosphorylation with LA/C [82,92].

The endosomal sorting complex required for transport (ESCRT)-III/vacuolar protein sorting-associated protein 4 (VPS4) is another machinery for resealing nuclear membranes during mitosis [93–96]. ESCRT-III forms a membrane-interacting oligomeric filament [97] and the AAA+ ATPase VPS4 drives the dynamic exchanges of ESCRT-III complex subunits for assembling and disassembling ESCRT-III filaments [98]. The LEM2 winged helix domain directly binds to an ESCRT-III subunit charged multivesicular body protein (CHMP) 7 [99], inducing the formation of a LEM2-CHMP7 copolymer [99]. At the sites where the membrane is intersected by microtubule bundles, LEM2 accumulates and undergoes liquid-phase separation, thereby triggering CHMP7 activation and ESCRT-III assembly. LEM2 recruits CHMP7 to the core region at telophase, followed by the sequential accumulation of other ESCRT-III complex subunits CHMP4B, CHMP2A and CHMP3 [93–96]. Coiled-coil- and C2 domain-containing protein 1B (CC2D1B) also accumulates at the core region in a CHMP7-dependent manner but it slows the accumulation of CHMP7, CHMP4B and CHMP2A [100]. CDK1 phosphorylates CHMP7 upon the entry of mitosis, which could prevent CHMP7 from interacting with LEM2 [96], suggesting that local CHMP7 dephosphorylation at the core region might license the LEM2-CHMP7 interaction that triggers ESCRT-III recruitment.

The tips of ER tubules could attach to mitotic chromosomes [101,102], supplying nuclear membranes containing B-type lamins, LBR and SUN1/2 to the peripheral chromosomal regions excluded from the core region at early telophase (the ‘non-core’ region) [62,74,93,94,103]. Nups are also localized to the non-core region, and this timely association of Nups could be regulated by a mechanism involving Importin β and RanGTP [104–106]. One of the first stages in NPC reassembly is the recruitment to the chromosome surface of ELYS [57,107,108]. ELYS interacts with the Nup107–160 complex and is essential for the recruitment of the Nup107–160 complex to chromatin [56,108,109].

The core region becomes gradually indistinguishable from the non-core region by the end of mitosis (~10 min) as the chromosomes decondense, establishing two distinct nuclei [10,74]. Concomitantly, lamins are dephosphorylated to form polymers in the NL [32,33,58,59]. A fraction of phosphorylated LA/C is transported into daughter nuclei at late telophase and diffusible throughout the nucleoplasm once the NE surrounds daughter chromosomes [61,74]. With the progression of the nuclear transport, the non-phosphorylated forms could become incorporated into the NL [110]. During the NE reformation, cytokinesis occurs through the construction of a contractile ring composed of actin and myosin filaments to separate into two daughter cells containing their own nuclei. When LA/C is expressed at high levels, the core region appears to remain in the area which are enriched in LA/C and devoid of NPCs during the following G1 phase of HeLa cells, designated as the ‘pore-free’ island [111].

## NE rupture

Mechanical stress can arise from external forces acting on the cell or from internal forces generated inside the cell itself. When these mechanical forces are excessive, the NE can locally burst with the immediate leakage of nuclear contents to the cytoplasm [112,113]. NE rupture occurs in pathological conditions including cancer cell migration [114,115]. Cancer cells often experience increased mechanical stress during migration, especially when passing through narrow spaces, such as blood vessels or tissue barriers. These mechanical forces can exert pressure on the nucleus and induce NE rupture [116]. Cancer cells secrete various proteolytic enzymes including matrix metalloproteinases (MMPs), which can degrade components of the extracellular matrix (ECM) to promote tumor growth in the early stages of tumorigenesis [117]. These enzymes can also facilitate cell migration leading to metastasis in the late stages [118], which could protect from NE rupture [114,119,120]. Internal forces generated by the cytoskeleton can exert mechanical stress on the NE through the LINC complex [121]. Disruptions in connections or alterations in the cytoskeletal dynamics can transmit mechanical forces directly to the NE, potentially resulting in NE rupture [112,122,123]. Numerous mutations that have been found throughout the A-type lamin gene cause a spectrum of human genetic disorders, correctively termed as the primary laminopathies [124]. It includes cardiac disorders characterized by dilated cardiomyopathy (DCM), hypertrophic cardiomyopathy (HCM), muscular dystrophy (MD), familial partial lipodystrophy (FPLD) and progeroid syndromes including Hutchinson–Gilford progeria syndrome (HGPS) [124]. Laminopathy mutations associated with DCM and MD frequently cause NE rupture in nuclear migration during skeletal muscle maturation [125]. When NE integrity is weakened by some of laminopathy mutations and lamin depletion, the NE can spontaneously rupture without an external force applied to the cell [126–132]. These phenotypes could be due to high curvature of the NE with locally diluted lamins in misshapen nuclei [133–135]. NE rupture can expose nuclear DNA to the cytoplasmic environment, which can potentially lead to DNA damage mediated by the ER-associated exonuclease 1 (TREX1) and activate DNA repair mechanisms [116]. DNA damage response proteins, such as ataxia telangiectasia mutated (ATM) and ataxia telangiectasia and Rad3-related (ATR), accumulates at the rupture sites to assess and repair any DNA damage that may have occurred [115,116,136]. The active form of a DNA sensor, cyclic GMP-AMP synthase (cGAS) in the cytoplasm binds to nuclear DNA at the opening area of the rupture sites [82,114,115,137] whereas the inactive form in the nucleus probably does not because of the preferential binding to histone H2A/H2B of nucleosomes [82,138–140]. The cytoplasmic cGAS binding to nuclear DNA can trigger immune responses and potentially lead to genomic instability and cell death through the signaling pathway with the downstream effector, stimulator of interferon genes (STING) [114,115].

## Repair of NE rupture

Upon the induction of NE rupture induced by mechanical stress, some of NE components are recruited to the rupture sites to help in sealing the holes or gaps and restoring its continuity [82,114,115,137,141]. BAF recruits A-type lamins and LEM-domain proteins to the rupture sites through the interactions with the Ig-fold domain and the LEM-domain, respectively [11,82,137,141–143], and the sequential order of the recruitment is similar to that of the accumulation at the core region. First, BAF rapidly accumulates at the rupture sites, which could be mediated by its ability to bind to nuclear DNA exposed to the cytoplasm [137]. It binds to exogenous dsDNA immediately after its appearance in the cytoplasm at endosome breakdown to form NE-like membranes to avoid autophagy [144] and outcompetes with cytoplasmic cGAS for binding nuclear DNA at the rupture sites to suppress an immune response through the cGAS-STING pathway [145]. However, BAF accumulation at the opening area of the rupture sites but not the DNA area might restrict the influx of cytoplasmic cGAS into the nucleus [82]. In addition, BAF is involved in DNA repair processes that may be triggered by NE rupture. Its binding to poly (ADP-ribose) polymerase 1 (PARP1) negatively regulates the DNA damage response to oxidative stress [146,147]. BAF also bind to DNA-dependent protein kinase catalytic subunit (DNA-PKcs) and directly inhibit the activity to increase non-homologous end joining [148]. BAF recruits LC from the nucleoplasm to the rupture sites and highly concentrate to form a relatively thick layer designated as the NE ‘plaque’ (within 1 min after the induction of NE rupture; Figure 2 and 3) [82,137]. Unlike the accumulation at the core region, LA is significantly slower (about 60 min) and weaker in localization to the NE plaque than LC because it is less abundant in the nucleoplasm than LC [82]. As with the accumulation at the core region, LA/C could target BAF to the NE plaque in reverse through BAF binding to the Ig-fold domain and BAF phosphorylation [74,82,82,92,141,142]. Then, LEM-domain proteins including LAP2α and β, Emerin, MAN1, LEM2 and Ankle2 accumulate at the NE plaque within 5 min (Figure 3) [137]. Because BAF phosphorylation suppresses its interaction with DNA but neither A-type lamins nor LEM-domain proteins [9], the phosphorylated form could recruit A-type lamins and LAP2α from the nucleoplasm to the NE plaque whereas LEM-domain containing INM proteins are brought from the cytoplasm with the non-phosphorylated form [8,10,137] (Figure 4). LB1 and LB2 are localized in later stages compared to LA and LC (Figure 3) [82], and NPCs are absent from the rupture sites. Because LB1 and several Nups in the central part of the NPC exhibit extremely low molecular turnovers and form immobile networks at the NE [149,150], the newly synthesized ones could slowly assemble at the rupture sites in interphase and/or their losses might not be completely recovered until they are recycled for NE reformation during mitosis.

As is the case with the recruitment to the core region, ESCRT-III and VPS4 are transiently recruited to the rupture site [114,115]. LEM2 recruits CHMP7 to the NE plaque [137], which probably directs CHMP4B, CHMP2A and CHMP3 to the edge of the NE plaque in a sequential order [114,115]. This LEM2-CHMP7 interaction can mediate the sealing of small holes (<100 nm) as LEM2-CHMP7 copolymers are formed around the diameter of ~100 nm *in vitro* [151], and ESCRTs are found in ~ 30–50 nm holes at the reforming NE [94]. On the other hand, BAF is involved in repairing much larger gaps at the rupture sites [82,114,115,137]. Just like the disassembly and reassembly of the NE in mitosis, the phosphorylation and dephosphorylation of NE components by specific kinases and phosphatases might be involved in repair of NE rupture, but the mechanisms remain poorly understood. The phosphorylation of LA could increase its nucleoplasmic pool to facilitate its rapid accumulation to the NE plaque [82,142,152,153]. The structure of the NE plaque remains for a while, depending on the size, and then gradually smooths out to become less prominent [82].

## Structure and functions of the core region and the NE plaque

In mitotic disassembly and interphase rupture of the NE, the partition between the nucleus and the cytoplasm is temporarily lost but these phenomena are very distinct from each other. For example, the NE completely disappears after NEBD whereas holes or gaps are locally made at the rupture sites. NE disassembly is not accompanied by DNA damage during normal segregation of mitotic chromosomes, but NE rupture clearly does. The disassembly is programed as a part of the cell-division processes. In contrast, the rupture can be randomly triggered by mechanical stress at any given time. Despite these differences, molecular mechanisms for mitotic NE reassembly and repair of NE rupture have some key features in common.

The core region and the NE plaque transiently emerge as precursor structures that are formed on the surface of chromatin and chromosomes with BAF and its downstream targets including A-type lamins, LEM-domain proteins and CHMPs, respectively (Figure 3). These structures appear to be stable because BAF is significantly immobile in the core region compared to that in the NE in interphase, forming a stable complex with LA and emerin [74,88], and LA is immobile in the NE plaque [142]. LEM2, as recruited to the core region with BAF, condenses into a liquid-like phase and forms a macromolecular O-ring with CHMP7 to seal nuclear membranes [99]. The O-ring structure could be also formed on the surface of the NE plaque to prevent the leakage of nuclear contents. Consequently, these dense structures might exclude B-type lamins and Nups from the core region and the NE plaque [74,82].

The thick layers of the core region and the NE plaque could temporally act as partition walls to prevent the unwanted interference of cytoplasmic components with chromatin (or mitotic chromosomes) until the resealing of nuclear membranes is complete [82,137,141]. The core region with a thickness of 24–64 nm that overlaps with the localization of BAF is clearly observed on the surface of the chromosome mass [74]. Even though the thickness of the NE plaque considerably depends on the size of holes or gaps, it could be in the range between ~76 nm and ~160 nm according to the recruitment of ESCRT-III complex subunits to the NE plaque [114]. This stopgap could be very effective for maintaining the functions of mitotic chromosomes and chromatin in interphase without interruption. The accumulation of BAF and its targets hardens the chromosome surface at the core region to block the penetration of spindle fibers into the chromosomes and cross-bridges the chromosomes to limit access of nuclear membranes to the surface [74,154,155]. These BAF-driven processes are critical for condensation and segregation of mitotic chromosomes and NE reassembly [8,76,156]. Formation of the NE plaque with BAF and its downstream targets could minimize the mixing of contents between the nucleus and the cytoplasm at the rupture sites to restore nuclear functions quickly [82,137,141].

## Concluding remarks

Evidence is accumulating to indicate that mitotic NE reassembly and repair of NE rupture are active areas of research, and our current understanding of the process is still evolving. Further studies are needed to uncover additional details and identify any variations in the restoration mechanisms under physiological and pathological conditions. Chromatin decondensation in G1 phase and DNA replication during interphase are coupled to a significant increase in nuclear size. The growing NE during these phases could be supplemented with the recycled and newly synthesized components by the mechanisms which could be similar to those that facilitate the mitotic reassembly and the repair. Dysregulation of these functions could be linked to some of laminopathy mutations and the BAF A12T mutation causing Néstor–Guillermo progeria syndrome (NGPS) [62,82,125,126,131,142,143,147,157].

## Data Availability

The image data included in this article is available from the corresponding author, TS, upon reasonable request.

## References

[1] Lammerding J, Schulze PC, Takahashi T, et al. Lamin A/C deficiency causes defective nuclear mechanics and mechanotransduction. J Clin Invest. 2004;113(3):370–15. doi: 10.1172/JCI200419670 14755334 PMC324542

[2] Shimi T, Pfleghaar K, Kojima S, et al. The A- and B-type nuclear lamin networks: microdomains involved in chromatin organization and transcription. Genes Dev. 2008;22(24):3409–3421. doi: 10.1101/gad.1735208 19141474 PMC2607069

[3] Levy DL, Heald R. Nuclear size Is regulated by importin α and Ntf2 in xenopus. Cell. 2010;143(2):288–298. doi: 10.1016/j.cell.2010.09.012 20946986 PMC2966892

[4] Swift J, Ivanovska IL, Buxboim A, et al. Nuclear lamin-A scales with tissue stiffness and enhances matrix-directed differentiation. Science. 2013;341(6149):1240104. doi: 10.1126/science.1240104 23990565 PMC3976548

[5] Shimi T, Kittisopikul M, Tran J, et al. Structural organization of nuclear lamins A, C, B1, and B2 revealed by superresolution microscopy. Mol Biol Cell. 2015;26(22):4075–4086. doi: 10.1091/mbc.E15-07-0461 26310440 PMC4710238

[6] Turgay Y, Eibauer M, Goldman AE, et al. The molecular architecture of lamins in somatic cells. Nature. 2017;543(7644):261–264. doi: 10.1038/nature21382 28241138 PMC5616216

[7] Foisner R, Gerace L. Integral membrane-proteins of the nuclear-envelope interact with lamins and chromosomes, and binding is modulated by mitotic phosphorylation. Cell. 1993;73(7):1267–1279. doi: 10.1016/0092-8674(93)90355-T 8324822

[8] Segura-Totten M, Kowalski AK, Craigie R, et al. Barrier-to-autointegration factor: major roles in chromatin decondensation and nuclear assembly. J Cell Bio. 2002;158(3):475–485. doi: 10.1083/jcb.200202019 12163470 PMC2173821

[9] Marcelot A, Petitalot A, Ropars V, et al. Di-phosphorylated BAF shows altered structural dynamics and binding to DNA, but interacts with its nuclear envelope partners. Nucleic Acids Res. 2021;49(7):3841–3855. doi: 10.1093/nar/gkab184 33744941 PMC8053085

[10] Haraguchi T, Koujin T, Segura-Totten M, et al. BAF is required for emerin assembly into the reforming nuclear envelope. J Cell Sci. 2001;114(24):4575–4585. doi: 10.1242/jcs.114.24.4575 11792822

[11] Samson C, Petitalot A, Celli F, et al. Structural analysis of the ternary complex between lamin A/C, BAF and emerin identifies an interface disrupted in autosomal recessive progeroid diseases. Nucleic Acids Res. 2018;46(19):10460–10473. doi: 10.1093/nar/gky736 30137533 PMC6212729

[12] Zullo JM, Demarco IA, Piqué-Regi R, et al. DNA sequence-dependent compartmentalization and silencing of chromatin at the nuclear lamina. Cell. 2012;149(7):1474–1487. doi: 10.1016/j.cell.2012.04.035 22726435

[13] Voeltz GK, Rolls MM, Rapoport TA. Structural organization of the endoplasmic reticulum. EMBO Rep. 2002;3(10):944–950. doi: 10.1093/embo-reports/kvf202 12370207 PMC1307613

[14] Zhen YY, Libotte T, Munck M, et al. NUANCE, a giant protein connecting the nucleus and actin cytoskeleton. J Cell Sci. 2002;115(15):3207–3222. doi: 10.1242/jcs.115.15.3207 12118075

[15] Libotte T, Zaim H, Abraham S, et al. Lamin A/C–dependent localization of nesprin-2, a giant scaffolder at the Nuclear Envelope. Mol Biol Cell. 2005;16(7):3411–3424. doi: 10.1091/mbc.e04-11-1009 15843432 PMC1165422

[16] Crisp M, Liu Q, Roux K, et al. Coupling of the nucleus and cytoplasm: role of the LINC complex. J Cell Bio. 2006;172(1):41–53. doi: 10.1083/jcb.200509124 16380439 PMC2063530

[17] Paine PL, Moore LC, Horowitz SB. Nuclear envelope permeability. Nature. 1975;254(5496):109–114. doi: 10.1038/254109a0 1117994

[18] Xie W, Chojnowski A, Boudier T, et al. A-type lamins form distinct filamentous networks with differential nuclear pore complex associations. Curr Biol. 2016;26(19):2651–2658. doi: 10.1016/j.cub.2016.07.049 27641764

[19] Kittisopikul M, Shimi T, Tatli M, et al. Computational analyses reveal spatial relationships between nuclear pore complexes and specific lamins. J Cell Bio. 2021;220(4):220. doi: 10.1083/jcb.202007082 PMC7883741 33570570

[20] Cronshaw JM, Krutchinsky AN, Zhang W, et al. Proteomic analysis of the mammalian nuclear pore complex. J Cell Bio. 2002;158(5):915–927. doi: 10.1083/jcb.200206106 12196509 PMC2173148

[21] Maimon T, Elad N, Dahan I, et al. The human nuclear pore complex as revealed by cryo-electron tomography. Structure. 2012;20(6):998–1006. doi: 10.1016/j.str.2012.03.025 22632834

[22] Bui KH, von Appen A, DiGuilio AL, et al. Integrated structural analysis of the human nuclear pore complex scaffold. Cell. 2013;155(6):1233–1243. doi: 10.1016/j.cell.2013.10.055 24315095

[23] von Appen A, Kosinski J, Sparks L, et al. In situ structural analysis of the human nuclear pore complex. Nature. 2015;526(7571):140–143. doi: 10.1038/nature15381 26416747 PMC4886846

[24] Mahamid J, Pfeffer S, Schaffer M, et al. Visualizing the molecular sociology at the HeLa cell nuclear periphery. Science. 2016;351(6276):969–972. doi: 10.1126/science.aad8857 26917770

[25] Schuller AP, Wojtynek M, Mankus D, et al. The cellular environment shapes the nuclear pore complex architecture. Nature. 2021;598(7882):667–671. doi: 10.1038/s41586-021-03985-3 34646014 PMC8550940

[26] Jacinto FV, Benner C, Hetzer MW. The nucleoporin Nup153 regulates embryonic stem cell pluripotency through gene silencing. Genes Dev. 2015;29(12):1224–1238. doi: 10.1101/gad.260919.115 26080816 PMC4495395

[27] Ibarra A, Benner C, Tyagi S, et al. Nucleoporin-mediated regulation of cell identity genes. Genes Dev. 2016;30(20):2253–2258. doi: 10.1101/gad.287417.116 27807035 PMC5110992

[28] Dey G, Baum B. Nuclear envelope remodelling during mitosis. Curr Opin Cell Biol. 2021;70:67–74. doi: 10.1016/j.ceb.2020.12.004 33421755 PMC8129912

[29] Yang L, Guan T, Gerace L. Integral membrane proteins of the nuclear envelope are dispersed throughout the endoplasmic reticulum during mitosis. J Cell Bio. 1997;137(6):1199–1210. doi: 10.1083/jcb.137.6.1199 9182656 PMC2132536

[30] Ellenberg J, Siggia ED, Moreira JE, et al. Nuclear membrane dynamics and reassembly in living cells: targeting of an inner nuclear membrane protein in interphase and mitosis. J Cell Bio. 1997;138(6):1193–1206. doi: 10.1083/jcb.138.6.1193 9298976 PMC2132565

[31] Puhka M, Vihinen H, Joensuu M, et al. Endoplasmic reticulum remains continuous and undergoes sheet-to-tubule transformation during cell division in mammalian cells. J Cell Bio. 2007;179(5):895–909. doi: 10.1083/jcb.200705112 18056408 PMC2099207

[32] Heald R, McKeon F. Mutations of phosphorylation sites in lamin a that prevent nuclear lamina disassembly in mitosis. Cell. 1990;61(4):579–589. doi: 10.1016/0092-8674(90)90470-Y 2344612

[33] Peter M, Nakagawa J, Dorée M, et al. In vitro disassembly of the nuclear lamina and M phase-specific phosphorylation of lamins by cdc2 kinase. Cell. 1990;61(4):591–602. doi: 10.1016/0092-8674(90)90471-P 2188731

[34] Hocevar BA, Burns DJ, Fields AP. Identification of protein kinase C (PKC) phosphorylation sites on human lamin B. Potential role of PKC in nuclear lamina structural dynamics. J Biol Chem. 1993;268(10):7545–7552. doi: 10.1016/S0021-9258(18)53210-5 8463284

[35] Goss VL, Hocevar BA, Thompson LJ, et al. Identification of nuclear beta II protein kinase C as a mitotic lamin kinase. J Biol Chem. 1994;269(29):19074–19080. doi: 10.1016/S0021-9258(17)32276-7 8034666

[36] Nichols RJ, Wiebe MS, Traktman P. The Vaccinia-related kinases phosphorylate the N' terminus of BAF, regulating its interaction with DNA and its retention in the nucleus. Mol Biol Cell. 2006;17(5):2451–2464. doi: 10.1091/mbc.e05-12-1179 16495336 PMC1446082

[37] Pfaller R, Smythe C, Newport JW. Assembly/Disassembly of the nuclear envelope membrane: cell cycle-dependent binding of nuclear membrane vesicles to chromatin in vitro. Cell. 1991;65(2):209–217. doi: 10.1016/0092-8674(91)90155-R 1849796

[38] Courvalin JC, Segil N, Blobel G, et al. The lamin B receptor of the inner nuclear membrane undergoes mitosis-specific phosphorylation and is a substrate for p34cdc2-type protein kinase. J Biol Chem. 1992;267(27):19035–19038. doi: 10.1016/S0021-9258(18)41734-6 1326541

[39] Olsen JV, Blagoev B, Gnad F, et al. Global, in vivo, and site-specific phosphorylation dynamics in signaling networks. Cell. 2006;127(3):635–648. doi: 10.1016/j.cell.2006.09.026 17081983

[40] Dephoure N, Zhou C, Villén J, et al. A quantitative atlas of mitotic phosphorylation. Proc Natl Acad Sci USA. 2008;105(31):10762–10767. doi: 10.1073/pnas.0805139105 18669648 PMC2504835

[41] Macaulay C, Meier E, Forbes DJ. Differential mitotic phosphorylation of proteins of the nuclear pore complex. J Biol Chem. 1995;270(1):254–262. doi: 10.1074/jbc.270.1.254 7814383

[42] Favreau C, Worman HJ, Wozniak RW, et al. Cell cycle-dependent phosphorylation of nucleoporins and nuclear pore membrane protein Gp210. Biochemistry. 1996;35(24):8035–8044. doi: 10.1021/bi9600660 8672508

[43] Laurell E, Beck K, Krupina K, et al. Phosphorylation of Nup98 by multiple kinases is crucial for NPC disassembly during mitotic entry. Cell. 2011;144(4):539–550. doi: 10.1016/j.cell.2011.01.012 21335236

[44] Linder MI, Köhler M, Boersema P, et al. Mitotic disassembly of nuclear pore complexes involves CDK1- and PLK1-mediated phosphorylation of key interconnecting nucleoporins. Dev Cell. 2017;43(2):141–56 e7. doi: 10.1016/j.devcel.2017.08.020 29065306 PMC5654724

[45] Güttinger S, Laurell E, Kutay U. Orchestrating nuclear envelope disassembly and reassembly during mitosis. Nat Rev Mol Cell Biol. 2009;10(3):178–191. doi: 10.1038/nrm2641 19234477

[46] Nurse P. Universal control mechanism regulating onset of M-phase. Nature. 1990;344(6266):503–508. doi: 10.1038/344503a0 2138713

[47] Hagting A, Jackman M, Simpson K, et al. Translocation of cyclin B1 to the nucleus at prophase requires a phosphorylation-dependent nuclear import signal. Curr Biol. 1999;9(13):680–689. doi: 10.1016/S0960-9822(99)80308-X 10395539

[48] Toyoshima-Morimoto F, Taniguchi E, Shinya N, et al. Polo-like kinase 1 phosphorylates cyclin B1 and targets it to the nucleus during prophase. Nature. 2001;410(6825):215–220. doi: 10.1038/35065617 11242082

[49] Gong D, Pomerening JR, Myers JW, et al. Cyclin A2 regulates nuclear-envelope breakdown and the nuclear accumulation of cyclin B1. Curr Biol. 2007;17(1):85–91. doi: 10.1016/j.cub.2006.11.066 17208191 PMC1830184

[50] Terasaki M, Campagnola P, Rolls MM, et al. A new model for nuclear envelope breakdown. Mol Biol Cell. 2001;12(2):503–510. doi: 10.1091/mbc.12.2.503 11179431 PMC30959

[51] Lénárt P, Rabut G, Daigle N, et al. Nuclear envelope breakdown in starfish oocytes proceeds by partial NPC disassembly followed by a rapidly spreading fenestration of nuclear membranes. J Cell Bio. 2003;160(7):1055–1068. doi: 10.1083/jcb.200211076 12654902 PMC2172766

[52] Terasaki M, Okumura E, Hinkle B, et al. Localization and dynamics of Cdc2-cyclin B during meiotic reinitiation in starfish oocytes. Mol Biol Cell. 2003;14(11):4685–4694. doi: 10.1091/mbc.e03-04-0249 14551249 PMC266783

[53] Salina D, Bodoor K, Eckley DM, et al. Cytoplasmic dynein as a facilitator of nuclear envelope breakdown. Cell. 2002;108(1):97–107. doi: 10.1016/S0092-8674(01)00628-6 11792324

[54] Georgatos SD, Pyrpasopoulou A, Theodoropoulos PA. Nuclear envelope breakdown in mammalian cells involves stepwise lamina disassembly and microtubule-drive deformation of the nuclear membrane. J Cell Sci. 1997;110(Pt 17):2129–2140. doi: 10.1242/jcs.110.17.2129 9378763

[55] Beaudouin J, Gerlich D, Daigle N, et al. Nuclear envelope breakdown proceeds by microtubule-induced tearing of the lamina. Cell. 2002;108(1):83–96. doi: 10.1016/S0092-8674(01)00627-4 11792323

[56] Glavy JS, Krutchinsky AN, Cristea IM, et al. Cell-cycle-dependent phosphorylation of the nuclear pore Nup107–160 subcomplex. Proc Natl Acad Sci USA. 2007;104(10):3811–3816. doi: 10.1073/pnas.0700058104 17360435 PMC1820666

[57] James C, Möller U, Spillner C, et al. Phosphorylation of ELYS promotes its interaction with VAPB at decondensing chromosomes during mitosis. EMBO Rep. 2024. doi: 10.1038/s44319-024-00125-6 PMC11094025 38605278

[58] Gerace L, Blobel G. The nuclear envelope lamina is reversibly depolymerized during mitosis. Cell. 1980;19(1):277–287. doi: 10.1016/0092-8674(80)90409-2 7357605

[59] Peter M, Heitlinger E, Häner M, et al. Disassembly of in vitro formed lamin head-to-tail polymers by CDC2 kinase. Embo J. 1991;10(6):1535–1544. doi: 10.1002/j.1460-2075.1991.tb07673.x 1851086 PMC452817

[60] Mall M, Walter T, Gorjánácz M, et al. Mitotic lamin disassembly is triggered by lipid-mediated signaling. J Cell Bio. 2012;198(6):981–990. doi: 10.1083/jcb.201205103 22986494 PMC3444782

[61] Moir RD, Yoon M, Khuon S, et al. Nuclear lamins a and B1: different pathways of assembly during nuclear envelope formation in living cells. J Cell Bio. 2000;151(6):1155–1168. doi: 10.1083/jcb.151.6.1155 11121432 PMC2190592

[62] Dechat T, Gajewski A, Korbei B, et al. LAP2α and BAF transiently localize to telomeres and specific regions on chromatin during nuclear assembly. J Cell Sci. 2004;117(25):6117–6128. doi: 10.1242/jcs.01529 15546916

[63] Kutay U, Jühlen R, Antonin W. Mitotic disassembly and reassembly of nuclear pore complexes. Trends Cell Biol. 2021;31(12):1019–1033. doi: 10.1016/j.tcb.2021.06.011 34294532

[64] Dohadwala M, da Cruz e Silva EF, Hall FL, et al. Phosphorylation and inactivation of protein phosphatase 1 by cyclin-dependent kinases. Proc Natl Acad Sci USA. 1994;91(14):6408–6412. doi: 10.1073/pnas.91.14.6408 8022797 PMC44211

[65] Kwon YG, Lee SY, Choi Y, et al. Cell cycle-dependent phosphorylation of mammalian protein phosphatase 1 by cdc2 kinase. Proc Natl Acad Sci USA. 1997;94(6):2168–2173. doi: 10.1073/pnas.94.6.2168 9122166 PMC20059

[66] Grallert A, Boke E, Hagting A, et al. A PP1–PP2A phosphatase relay controls mitotic progression. Nature. 2015;517(7532):94–98. doi: 10.1038/nature14019 25487150 PMC4338534

[67] Murray AW, Solomon MJ, Kirschner MW. The role of cyclin synthesis and degradation in the control of maturation promoting factor activity. Nature. 1989;339(6222):280–286. doi: 10.1038/339280a0 2566918

[68] Skoufias DA, Indorato RL, Lacroix F, et al. Mitosis persists in the absence of Cdk1 activity when proteolysis or protein phosphatase activity is suppressed. J Cell Bio. 2007;179(4):671–685. doi: 10.1083/jcb.200704117 18025303 PMC2080905

[69] Thompson LJ, Bollen M, Fields AP. Identification of protein phosphatase 1 as a mitotic lamin phosphatase. J Biol Chem. 1997;272(47):29693–29697. doi: 10.1074/jbc.272.47.29693 9368037

[70] Steen RL, Martins SB, Taskén K, et al. Recruitment of protein phosphatase 1 to the nuclear envelope by A-kinase anchoring protein AKAP149 is a prerequisite for nuclear lamina assembly. J Cell Bio. 2000;150(6):1251–1262. doi: 10.1083/jcb.150.6.1251 10995432 PMC2150688

[71] Steen RL, Beullens M, Landsverk HB, et al. AKAP149 is a novel PP1 specifier required to maintain nuclear envelope integrity in G1 phase. J Cell Sci. 2003;116(11):2237–2246. doi: 10.1242/jcs.00432 12697839

[72] Trinkle-Mulcahy L, Andersen J, Lam YW, et al. Repo-Man recruits PP1γ to chromatin and is essential for cell viability. J Cell Bio. 2006;172(5):679–692. doi: 10.1083/jcb.200508154 16492807 PMC2063701

[73] Vagnarelli P, Ribeiro S, Sennels L, et al. Repo-Man coordinates chromosomal reorganization with nuclear envelope reassembly during mitotic exit. Dev Cell. 2011;21(2):328–342. doi: 10.1016/j.devcel.2011.06.020 21820363 PMC3480639

[74] Haraguchi T, Kojidani T, Koujin T, et al. Live cell imaging and electron microscopy reveal dynamic processes of BAF-directed nuclear envelope assembly. J Cell Sci. 2008;121(15):2540–2554. doi: 10.1242/jcs.033597 18628300

[75] Asencio C, Davidson IF, Santarella-Mellwig R, et al. Coordination of kinase and phosphatase activities by Lem4 enables nuclear envelope reassembly during mitosis. Cell. 2012;150(1):122–135. doi: 10.1016/j.cell.2012.04.043 22770216

[76] Zheng R, Ghirlando R, Lee MS, et al. Barrier-to-autointegration factor (BAF) bridges DNA in a discrete, higher-order nucleoprotein complex. Proc Natl Acad Sci USA. 2000;97(16):8997–9002. doi: 10.1073/pnas.150240197 10908652 PMC16810

[77] Harris D, Engelman A. Both the structure and DNA binding function of the barrier-to-autointegration factor contribute to reconstitution of HIV type 1 integration in vitro. J Biol Chem. 2000;275(50):39671–39677. doi: 10.1074/jbc.M002626200 11005805

[78] Lee KK, Haraguchi T, Lee RS, et al. Distinct functional domains in emerin bind lamin a and DNA-bridging protein BAF. J Cell Sci. 2001;114(24):4567–4573. doi: 10.1242/jcs.114.24.4567 11792821

[79] Bradley CM, Ronning DR, Ghirlando R, et al. Structural basis for DNA bridging by barrier-to-autointegration factor. Nat Struct Mol Biol. 2005;12(10):935–936. doi: 10.1038/nsmb989 16155580

[80] Zhuang X, Semenova E, Maric D, et al. Dephosphorylation of barrier-to-autointegration factor by protein phosphatase 4 and its role in cell mitosis. J Biol Chem. 2014;289(2):1119–1127. doi: 10.1074/jbc.M113.492777 24265311 PMC3887179

[81] Gorjánácz M, Klerkx EP, Galy V, et al. Caenorhabditis elegans BAF-1 and its kinase VRK-1 participate directly in post-mitotic nuclear envelope assembly. Embo J. 2007;26(1):132–143. doi: 10.1038/sj.emboj.7601470 17170708 PMC1782363

[82] Kono Y, Adam SA, Sato Y, et al. Nucleoplasmic lamin C rapidly accumulates at sites of nuclear envelope rupture with BAF and cGAS. J Cell Bio. 2022;221(12):221. doi: 10.1083/jcb.202201024 PMC9617480 36301259

[83] Furukawa K. LAP2 binding protein 1 (L2BP1/BAF) is a candidate mediator of LAP2-chromatin interaction. J Cell Sci. 1999;112(Pt 15):2485–2492. doi: 10.1242/jcs.112.15.2485 10393804

[84] Lin F, Blake DL, Callebaut I, et al. MAN1, an inner nuclear membrane protein that shares the LEM domain with lamina-associated polypeptide 2 and emerin. J Biol Chem. 2000;275(7):4840–4847. doi: 10.1074/jbc.275.7.4840 10671519

[85] Dechat T, Korbei B, Vaughan OA, et al. Lamina-associated polypeptide 2alpha binds intranuclear A-type lamins. J Cell Sci. 2000;113(Pt 19):3473–3484. doi: 10.1242/jcs.113.19.3473 10984438

[86] Shumaker DK, Lee KK, Tanhehco YC, et al. LAP2 binds to BAF·DNA complexes: requirement for the LEM domain and modulation by variable regions. Embo J. 2001;20(7):1754–1764. doi: 10.1093/emboj/20.7.1754 11285238 PMC145505

[87] Cai M, Huang Y, Ghirlando R, et al. Solution structure of the constant region of nuclear envelope protein LAP2 reveals two LEM-domain structures: one binds BAF and the other binds DNA. Embo J. 2001;20(16):4399–4407. doi: 10.1093/emboj/20.16.4399 11500367 PMC125263

[88] Shimi T, Koujin T, Segura-Totten M, et al. Dynamic interaction between BAF and emerin revealed by FRAP, FLIP, and FRET analyses in living HeLa cells. J Struct Biol. 2004;147(1):31–41. doi: 10.1016/j.jsb.2003.11.013 15109603

[89] Torras-Llort M, Medina-Giró S, Escudero-Ferruz P, et al. A fraction of barrier-to-autointegration factor (BAF) associates with centromeres and controls mitosis progression. Commun Biol. 2020;3(1):454. doi: 10.1038/s42003-020-01182-y 32814801 PMC7438335

[90] Mehsen H, Boudreau V, Garrido D, et al. PP2A-B55 promotes nuclear envelope reformation after mitosis in Drosophila. J Cell Bio. 2018;217(12):4106–4123. doi: 10.1083/jcb.201804018 30309980 PMC6279390

[91] Haraguchi T, Koujin T, Osakada H, et al. Nuclear localization of barrier-to-autointegration factor is correlated with progression of S phase in human cells. J Cell Sci. 2007;120(12):1967–1977. doi: 10.1242/jcs.03461 17519288

[92] Lin Q, Yu B, Wang X, et al. K6-linked SUMOylation of BAF regulates nuclear integrity and DNA replication in mammalian cells. Proc Natl Acad Sci USA. 2020;117(19):10378–10387. doi: 10.1073/pnas.1912984117 32332162 PMC7229763

[93] Vietri M, Schink KO, Campsteijn C, et al. Spastin and ESCRT-III coordinate mitotic spindle disassembly and nuclear envelope sealing. Nature. 2015;522(7555):231–235. doi: 10.1038/nature14408 26040712

[94] Olmos Y, Hodgson L, Mantell J, et al. ESCRT-III controls nuclear envelope reformation. Nature. 2015;522(7555):236–239. doi: 10.1038/nature14503 26040713 PMC4471131

[95] Gu M, LaJoie D, Chen OS, et al. LEM2 recruits CHMP7 for ESCRT-mediated nuclear envelope closure in fission yeast and human cells. Proc Natl Acad Sci USA. 2017;114(11):E2166–E75. doi: 10.1073/pnas.1613916114 28242692 PMC5358359

[96] Gatta AT, Olmos Y, Stoten CL, et al. CDK1 controls CHMP7-dependent nuclear envelope reformation. Elife. 2021;10:10. doi: 10.7554/eLife.59999 PMC8324300 34286694

[97] Schöneberg J, Pavlin MR, Yan S, et al. ATP-dependent force generation and membrane scission by ESCRT-III and Vps4. Science. 2018;362(6421):1423–1428. doi: 10.1126/science.aat1839 30573630 PMC6309985

[98] Mierzwa BE, Chiaruttini N, Redondo-Morata L, et al. Dynamic subunit turnover in ESCRT-III assemblies is regulated by Vps4 to mediate membrane remodelling during cytokinesis. Nat Cell Biol. 2017;19(7):787–798. doi: 10.1038/ncb3559 28604678 PMC5493987

[99] von Appen A, LaJoie D, Johnson IE, et al. LEM2 phase separation promotes ESCRT-mediated nuclear envelope reformation. Nature. 2020;582(7810):115–118. doi: 10.1038/s41586-020-2232-x 32494070 PMC7321842

[100] Ventimiglia LN, Cuesta-Geijo MA, Martinelli N, et al. CC2D1B coordinates ESCRT-III activity during the mitotic reformation of the nuclear envelope. Dev Cell. 2018;47(5):547–563 e6. doi: 10.1016/j.devcel.2018.11.012 30513301 PMC6286407

[101] Anderson DJ, Hetzer MW. Nuclear envelope formation by chromatin-mediated reorganization of the endoplasmic reticulum. Nat Cell Biol. 2007;9(10):1160–1166. doi: 10.1038/ncb1636 17828249

[102] Anderson DJ, Hetzer MW. Shaping the endoplasmic reticulum into the nuclear envelope. J Cell Sci. 2008;121(2):137–142. doi: 10.1242/jcs.005777 18187447

[103] Chi YH, Haller K, Peloponese JM Jr., et al. Histone acetyltransferase hALP and nuclear membrane protein hsSUN1 function in de-condensation of mitotic chromosomes. J Biol Chem. 2007;282(37):27447–27458. doi: 10.1074/jbc.M703098200 17631499

[104] Kutay U, Bischoff FR, Kostka S, et al. Export of importin α from the nucleus Is mediated by a specific nuclear transport factor. Cell. 1997;90(6):1061–1071. doi: 10.1016/S0092-8674(00)80372-4 9323134

[105] Walther TC, Askjaer P, Gentzel M, et al. RanGTP mediates nuclear pore complex assembly. Nature. 2003;424(6949):689–694. doi: 10.1038/nature01898 12894213

[106] Harel A, Chan RC, Lachish-Zalait A, et al. Importin β negatively regulates nuclear membrane fusion and nuclear pore complex assembly. Mol Biol Cell. 2003;14(11):4387–4396. doi: 10.1091/mbc.e03-05-0275 14551248 PMC266759

[107] Franz C, Walczak R, Yavuz S, et al. MEL-28/ELYS is required for the recruitment of nucleoporins to chromatin and postmitotic nuclear pore complex assembly. EMBO Rep. 2007;8(2):165–172. doi: 10.1038/sj.embor.7400889 17235358 PMC1796766

[108] Rasala BA, Ramos C, Harel A, et al. Capture of AT-rich chromatin by ELYS recruits POM121 and NDC1 to initiate nuclear pore assembly. Mol Biol Cell. 2008;19(9):3982–3996. doi: 10.1091/mbc.e08-01-0012 18596237 PMC2526682

[109] Rasala BA, Orjalo AV, Shen Z, et al. ELYS is a dual nucleoporin/kinetochore protein required for nuclear pore assembly and proper cell division. Proc Natl Acad Sci USA. 2006;103(47):17801–17806. doi: 10.1073/pnas.0608484103 17098863 PMC1635652

[110] Yang L, Guan T, Gerace L. Lamin-binding fragment of LAP2 inhibits increase in nuclear volume during the cell cycle and progression into S phase. J Cell Bio. 1997;139(5):1077–1087. doi: 10.1083/jcb.139.5.1077 9382857 PMC2140217

[111] Maeshima K, Yahata K, Sasaki Y, et al. Cell-cycle-dependent dynamics of nuclear pores: pore-free islands and lamins. J Cell Sci. 2006;119(21):4442–4451. doi: 10.1242/jcs.03207 17074834

[112] Hatch EM, Hetzer MW. Nuclear envelope rupture is induced by actin-based nucleus confinement. J Cell Bio. 2016;215(1):27–36. doi: 10.1083/jcb.201603053 27697922 PMC5057282

[113] Zhang Q, Tamashunas AC, Agrawal A, et al. Local, transient tensile stress on the nuclear membrane causes membrane rupture. Mol Biol Cell. 2019;30(7):899–906. doi: 10.1091/mbc.E18-09-0604 30566037 PMC6589786

[114] Denais CM, Gilbert RM, Isermann P, et al. Nuclear envelope rupture and repair during cancer cell migration. Science. 2016;352(6283):353–358. doi: 10.1126/science.aad7297 27013428 PMC4833568

[115] Raab M, Gentili M, de Belly H, et al. ESCRT III repairs nuclear envelope ruptures during cell migration to limit DNA damage and cell death. Science. 2016;352(6283):359–362. doi: 10.1126/science.aad7611 27013426

[116] Nader GPF, Agüera-Gonzalez S, Routet F, et al. Compromised nuclear envelope integrity drives TREX1-dependent DNA damage and tumor cell invasion. Cell. 2021;184(20):5230–46 e22. doi: 10.1016/j.cell.2021.08.035 34551315

[117] Liotta LA, Tryggvason K, Garbisa S, et al. Metastatic potential correlates with enzymatic degradation of basement membrane collagen. Nature. 1980;284(5751):67–68. doi: 10.1038/284067a0 6243750

[118] Wolf K, Wu YI, Liu Y, et al. Multi-step pericellular proteolysis controls the transition from individual to collective cancer cell invasion. Nat Cell Biol. 2007;9(8):893–904. doi: 10.1038/ncb1616 17618273

[119] Wolf K, Te Lindert M, Krause M, et al. Physical limits of cell migration: control by ECM space and nuclear deformation and tuning by proteolysis and traction force. J Cell Bio. 2013;201(7):1069–1084. doi: 10.1083/jcb.201210152 23798731 PMC3691458

[120] Infante E, Castagnino A, Ferrari R, et al. LINC complex-Lis1 interplay controls MT1-MMP matrix digest-on-demand response for confined tumor cell migration. Nat Commun. 2018;9(1):2443. doi: 10.1038/s41467-018-04865-7 29934494 PMC6015082

[121] Kalukula Y, Stephens AD, Lammerding J, et al. Mechanics and functional consequences of nuclear deformations. Nat Rev Mol Cell Biol. 2022;23(9):583–602. doi: 10.1038/s41580-022-00480-z 35513718 PMC9902167

[122] Patteson AE, Vahabikashi A, Pogoda K, et al. Vimentin protects cells against nuclear rupture and DNA damage during migration. J Cell Bio. 2019;218(12):4079–4092. doi: 10.1083/jcb.201902046 31676718 PMC6891099

[123] Thiam HR, Wong SL, Qiu R, et al. Netosis proceeds by cytoskeleton and endomembrane disassembly and PAD4-mediated chromatin decondensation and nuclear envelope rupture. Proc Natl Acad Sci USA. 2020;117(13):7326–7337. doi: 10.1073/pnas.1909546117 32170015 PMC7132277

[124] Worman HJ, Bonne G. “Laminopathies”: a wide spectrum of human diseases. Exp Cell Res. 2007;313(10):2121–2133. doi: 10.1016/j.yexcr.2007.03.028 17467691 PMC2964355

[125] Earle AJ, Kirby TJ, Fedorchak GR, et al. Mutant lamins cause nuclear envelope rupture and DNA damage in skeletal muscle cells. Nat Mater. 2020;19(4):464–473. doi: 10.1038/s41563-019-0563-5 31844279 PMC7102937

[126] De Vos WH, Houben F, Kamps M, et al. Repetitive disruptions of the nuclear envelope invoke temporary loss of cellular compartmentalization in laminopathies. Hum Mol Genet. 2011;20(21):4175–4186. doi: 10.1093/hmg/ddr344 21831885

[127] Vargas JD, Hatch EM, Anderson DJ, et al. Transient nuclear envelope rupturing during interphase in human cancer cells. Nucleus. 2012;3(1):88–100. doi: 10.4161/nucl.18954 22567193 PMC3342953

[128] Robijns J, Molenberghs F, Sieprath T, et al. In silico synchronization reveals regulators of nuclear ruptures in lamin A/C deficient model cells. Sci Rep. 2016;6(1):30325. doi: 10.1038/srep30325 27461848 PMC4962089

[129] Chen NY, Kim P, Weston TA, et al. Fibroblasts lacking nuclear lamins do not have nuclear blebs or protrusions but nevertheless have frequent nuclear membrane ruptures. Proc Natl Acad Sci USA. 2018;115(40):10100–10105. doi: 10.1073/pnas.1812622115 30224463 PMC6176609

[130] Chen NY, Yang Y, Weston TA, et al. An absence of lamin B1 in migrating neurons causes nuclear membrane ruptures and cell death. Proc Natl Acad Sci USA. 2019;116(51):25870–25879. doi: 10.1073/pnas.1917225116 31796586 PMC6926041

[131] Kim PH, Chen NY, Heizer PJ, et al. Nuclear membrane ruptures underlie the vascular pathology in a mouse model of Hutchinson-Gilford progeria syndrome. JCI Insight. 2021;6(16):6. doi: 10.1172/jci.insight.151515 PMC8409987 34423791

[132] Vahabikashi A, Sivagurunathan S, Nicdao FAS, et al. Nuclear lamin isoforms differentially contribute to LINC complex-dependent nucleocytoskeletal coupling and whole-cell mechanics. Proc Natl Acad Sci USA. 2022;119(17):e2121816119. doi: 10.1073/pnas.2121816119 35439057 PMC9170021

[133] Xia Y, Ivanovska IL, Zhu K, et al. Nuclear rupture at sites of high curvature compromises retention of DNA repair factors. J Cell Bio. 2018;217(11):3796–3808. doi: 10.1083/jcb.201711161 30171044 PMC6219729

[134] Xia Y, Pfeifer CR, Zhu K, et al. Rescue of DNA damage after constricted migration reveals a mechano-regulated threshold for cell cycle. J Cell Bio. 2019;218(8):2545–2563. doi: 10.1083/jcb.201811100 31239284 PMC6683732

[135] Ivanovska IL, Tobin MP, Bai T, et al. Small lipid droplets are rigid enough to indent a nucleus, dilute the lamina, and cause rupture. J Cell Bio. 2023;222(8):222. doi: 10.1083/jcb.202208123 PMC10202833 37212777

[136] Kidiyoor GR, Li Q, Bastianello G, et al. ATR is essential for preservation of cell mechanics and nuclear integrity during interstitial migration. Nat Commun. 2020;11(1):4828. doi: 10.1038/s41467-020-18580-9 32973141 PMC7518249

[137] Halfmann CT, Sears RM, Katiyar A, et al. Repair of nuclear ruptures requires barrier-to-autointegration factor. J Cell Bio. 2019;218(7):2136–2149. doi: 10.1083/jcb.201901116 31147383 PMC6605789

[138] Pathare GR, Decout A, Glück S, et al. Structural mechanism of cGAS inhibition by the nucleosome. Nature. 2020;587(7835):668–672. doi: 10.1038/s41586-020-2750-6 32911482

[139] Zhao B, Xu P, Rowlett CM, et al. The molecular basis of tight nuclear tethering and inactivation of cGAS. Nature. 2020;587(7835):673–677. doi: 10.1038/s41586-020-2749-z 32911481 PMC7704945

[140] Kujirai T, Zierhut C, Takizawa Y, et al. Structural basis for the inhibition of cGAS by nucleosomes. Science. 2020;370(6515):455–458. doi: 10.1126/science.abd0237 32912999 PMC7584773

[141] Young AM, Gunn AL, Hatch EM, et al. BAF facilitates interphase nuclear membrane repair through recruitment of nuclear transmembrane proteins. Mol Biol Cell. 2020;31(15):1551–1560. doi: 10.1091/mbc.E20-01-0009 32459568 PMC7521799

[142] Sears RM, Roux KJ. Mechanisms of A-type lamin targeting to nuclear ruptures are disrupted in LMNA- and BANF1-associated progerias. Cells. 2022:11(5): 865. doi: 10.3390/cells11050865 35269487 PMC8909658

[143] Janssen A, Marcelot A, Breusegem S, et al. The BAF A12T mutation disrupts lamin A/C interaction, impairing robust repair of nuclear envelope ruptures in nestor–Guillermo progeria syndrome cells. Nucleic Acids Res. 2022;50(16):9260–9278. doi: 10.1093/nar/gkac726 36039758 PMC9458464

[144] Kobayashi S, Koujin T, Kojidani T, et al. BAF is a cytosolic DNA sensor that leads to exogenous DNA avoiding autophagy. Proc Natl Acad Sci USA. 2015;112(22):7027–7032. doi: 10.1073/pnas.1501235112 25991860 PMC4460496

[145] Guey B, Wischnewski M, Decout A, et al. BAF restricts cGAS on nuclear DNA to prevent innate immune activation. Science. 2020;369(6505):823–828. doi: 10.1126/science.aaw6421 32792394

[146] Montes de Oca R, Shoemaker CJ, Gucek M, et al. Barrier-to-autointegration factor proteome reveals chromatin-regulatory partners. PLoS One. 2009;4(9):e7050. doi: 10.1371/journal.pone.0007050 19759913 PMC2739719

[147] Bolderson E, Burgess JT, Li J, et al. Barrier-to-autointegration factor 1 (Banf1) regulates poly [ADP-ribose] polymerase 1 (PARP1) activity following oxidative DNA damage. Nat Commun. 2019;10(1):5501. doi: 10.1038/s41467-019-13167-5 31796734 PMC6890647

[148] Burgess JT, Cheong CM, Suraweera A, et al. Barrier-to-autointegration-factor (Banf1) modulates DNA double-strand break repair pathway choice via regulation of DNA-dependent kinase (DNA-PK) activity. Nucleic Acids Res. 2021;49(6):3294–3307. doi: 10.1093/nar/gkab110 33660778 PMC8034644

[149] Daigle N, Beaudouin J, Hartnell L, et al. Nuclear pore complexes form immobile networks and have a very low turnover in live mammalian cells. J Cell Bio. 2001;154(1):71–84. doi: 10.1083/jcb.200101089 11448991 PMC2196857

[150] Rabut G, Doye V, Ellenberg J. Mapping the dynamic organization of the nuclear pore complex inside single living cells. Nat Cell Biol. 2004;6(11):1114–1121. doi: 10.1038/ncb1184 15502822

[151] Vietri M, Schultz SW, Bellanger A, et al. Unrestrained ESCRT-III drives micronuclear catastrophe and chromosome fragmentation. Nat Cell Biol. 2020;22(7):856–867. doi: 10.1038/s41556-020-0537-5 32601372

[152] Kochin V, Shimi T, Torvaldson E, et al. Interphase phosphorylation of lamin A. J Cell Sci. 2014;127:2683–2696. doi: 10.1242/jcs.141820 24741066 PMC4058112

[153] Cho S, Abbas A, Irianto J, et al. Progerin phosphorylation in interphase is lower and less mechanosensitive than lamin-A,C in iPS-derived mesenchymal stem cells. Nucleus. 2018;9(1):230–245. doi: 10.1080/19491034.2018.1460185 29619860 PMC5973135

[154] Samwer M, Schneider MWG, Hoefler R, et al. DNA cross-bridging shapes a single nucleus from a set of mitotic chromosomes. Cell. 2017;170(5):956–972 e23. doi: 10.1016/j.cell.2017.07.038 28841419 PMC5638020

[155] Schneider MWG, Gibson BA, Otsuka S, et al. A mitotic chromatin phase transition prevents perforation by microtubules. Nature. 2022;609(7925):183–190. doi: 10.1038/s41586-022-05027-y 35922507 PMC9433320

[156] Margalit A, Vlcek S, Gruenbaum Y, et al. Breaking and making of the nuclear envelope. J Cell Biochem. 2005;95(3):454–465. doi: 10.1002/jcb.20433 15832341

[157] Dechat T, Shimi T, Adam SA, et al. Alterations in mitosis and cell cycle progression caused by a mutant lamin a known to accelerate human aging. Proc Natl Acad Sci USA. 2007;104(12):4955–4960. doi: 10.1073/pnas.0700854104 17360326 PMC1829246

